# Cotton-Wool Sandwiches

**Published:** 1902-04-05

**Authors:** 


### COTTON-WOOL SANDWICHES.

A very curious case was reported to a recent meeting of the Royal Academy of Medicine in Ireland by Dr. Jameson Johnston. A young man, 20 years of age, whilst swimming in the sea was struck in the face by a wave. He at once felt a choking sensation, but was able to get to shore. He told the bystanders that he was being choked by his false teeth, and they slapped him on the back with the object of relieving him. This at first produced dyspnœa, but a further slapping removed this, though it caused him much pain. On admission to hospital a probang was passed, and so much relief was experienced that it was thought that the denture had passed into the stomach, an opinion which was confirmed by the fact that soon afterwards pains were experienced at the pyloric end of the stomach and continued for some days. On this Mr. Johnston decided on feeding him with sandwiches each containing a thin layer of cotton-wool. A week after admission a dose of compound liquorice powder was given, and, on this acting, the denture, it is stated, was found in the fæces, rolled up in cotton wool!

